# Le chondrome extra osseux de la cuisse: une localisation exceptionnelle (à propos d'un cas)

**DOI:** 10.11604/pamj.2015.21.44.6772

**Published:** 2015-05-21

**Authors:** Bouchaib Chafry, Youness Sasbou, Mohammed Boussaidane, Mustafa Nkaoui, Driss Benchebba, Belkacem Chagar

**Affiliations:** 1 Service de Traumatologie-Orthopédie, Hôpital Militaire Mohamed V, Rabat, Maroc

**Keywords:** Chondrome, extra osseux, cuisse, Chondroma, extra bone, thigh

## Abstract

Le chondrome extra-osseux est une tumeur cartilagineuse bénigne et rare. Il siège généralement au niveau des extrémités, nous rapportons un cas exceptionnel d'un chondrome des parties molles de la cuisse chez un homme de 35 ans qui s'est manifesté par une tuméfaction douloureuse de la face interne de la cuisse droite. L'histologie a confirmé le diagnostic sur la pièce d'exérèse. Un suivi clinique et radiologique après un recul de 24 mois n'a pas objectivé de signe de récidive.

## Introduction

Le chondrome est une tumeur bénigne fréquente siégeant généralement au niveau osseux. Sa localisation extra osseuse est extrêmement rare [1, 2]. Le chondrome des parties molles siège préférentiellement à la main ou au pied, son évolution est lente [2, 3]. Il est formée de nodules cartilagineux bien limités développés au sein des parties molles, sans adhésion à l'os ou au périoste ce qui le différencie du chondrome juxtacortical ou périosté. Nous rapportons un cas exceptionnel de chondrome extra-osseux de la cuisse, en mettant l'accent sur les signes cliniques, radiologiques et histologiques avec une revue de littérature.

## Patient et observation

**Figure 1:**
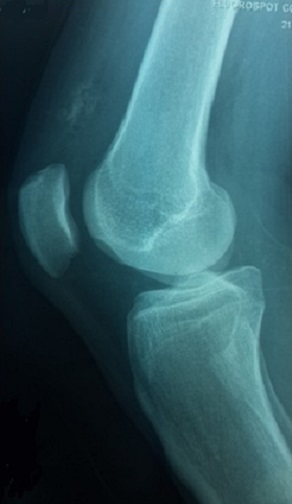
Radiographie standard en incidence de profil du genou droit montrant la masse en tonalité calcique

**Figure 2:**
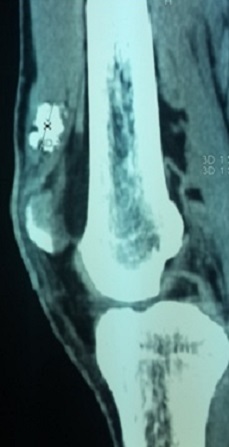
TDM en coupe sagittale montrant la localisation de la tumeur

**Figure 3:**
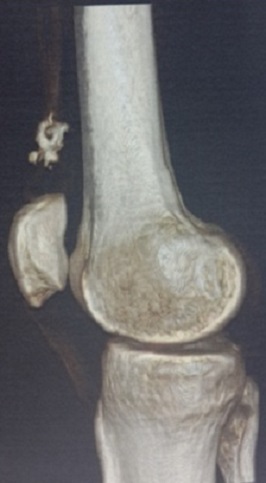
TDM en reconstruction 3D

**Figure 4:**
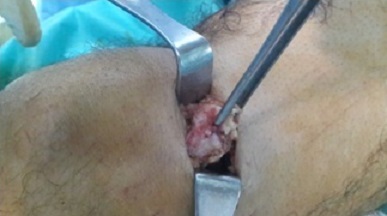
Image per-opératoire objectivant la tumeur

**Figure 5:**
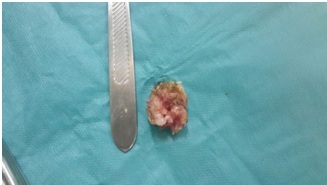
Image per-opératoire de la pièce opératoire

Il s'agit d'un homme âgé de 35 ans qui consulte pour une tuméfaction douloureuse de la face interne de la cuisse droite, apparue deux ans auparavant et augmentant lentement de volume, sans notion de traumatisme ancien ni d'antécédents médicaux ou chirurgicaux à l'interrogatoire. L'examen physique a objectivé une tuméfaction visible, siégeant à la face interne du tiers inferieur de la cuisse droite, ferme mobile et indolore à la palpation, sans signes cutanées associées. L'examen du membre inferieur droit et le reste de l'examen physique ne trouvait pas d'anomalies. Les radiographies de la cuisse droite ont objectivées une image de tonalité calcique de 25 mm de diamètre, siégeant dans les parties molles, sans attache au périoste (Figure 1). La tomodensitométrie a montré une image de densité calcique siégeant dans le corps musculaire du vaste médial en extra articulaire sans contact avec l'os ou avec l'articulation du genou (Figure 2). L'exploration chirurgicale a révélé une tumeur encapsulée adhérente aux fibres du muscle vaste médial. Après une dissection, l'excision complète a pu être réalisée (Figure 3). Il s'agissait d'une masse calcifiée, blanchâtre, lobulée, de consistance ferme (Figure 4). L'examen macroscopique a révélé une tumeur lobulée fortement calcifiée alors que l'aspect histologique microscopique Une biopsie chirurgicale a été réalisée. L'aspect histologique était en faveur d'une tumeur chondroblastique avec une cellularité modérée correspondant à un chondrome (Figure 5). Après un recul de 24 mois, le malade est asymptomatique sans aucun signe de récidive à l'IRM.

## Discussion

Les chondromes extra-osseux sont relativement rares, représentant environ 1,5% de l'ensemble des tumeurs bénignes des parties molles [4]. Ils peuvent survenir à tout âge mais sont surtout fréquents entre la troisième et la septième décennie [4, 5], généralement situées au contact des tissus péri-articulaires ou des gaines tendineuses, sans adhésions à l'os [6–8], ils siègent presque toujours dans les extrémités, souvent aux mains [6]. Aucun cas de chondrome extra-osseux de la cuisse n'a été rapporté dans la littérature. L’étiologie de ces tumeurs reste dans la plupart des cas non déterminée, il s'agirait d'une prolifération de la synoviale et certains auteurs en rapprochent la chondromatose synoviale, chondromatose qui touche généralement les grosses articulations [9]. La tumeur se développe habituellement dans une extrémité, souvent près d'un tendon ou d'une gaine tendineuse, d'une capsule articulaire ou du périoste [4]. Chez notre malade, la masse se situait près du tendon du vaste medial. Sur le plan clinique, il s'agit d'une tuméfaction indolore des parties molles, augmentant lentement de volume, de sorte que le malade ne consulte habituellement que tardivement. [10, 11]. Sur le plan radiologique, L'aspect du chondrome extra-osseux varie selon l'importance de la calcification du foyer tumoral. Un foyer de calcification se développe dans environ un tiers de cas, habituellement au centre de la lésion [3, 8].

Bien que le chondrome extra osseux ne soit pas attaché à l'os, il peut provoquer un remaniement du cortex adjacent [4]. L'exploration radiologique a mis en évidence chez notre malade une masse calcifiée située dans les parties molles, sans contact avec l'os. Sur le plan histologique, l'examen macroscopique montre habituellement une tumeur lobulée, bien encapsulée, caoutchouteuse [8]. Tandis que L'examen microscopique montre la présence de chondrocytes marqués par l'anticorps antiprotéine S100 [12]. L'examen au microscope électronique montre des chondrocytes contenant de grands noyaux dentelés, un réticulum endoplasmique rugueux abondant et par endroits des vacuoles attachées à la membrane. De courtes microvillosités ou filopodes s’étendent de la membrane cellulaire dans la substance fondamentale avoisinante. Cette dernière contient, en cas de tumeur calcifiée, des agrégats de cristaux d'hydroxyapatite de taille variable [8]. L'excision chirurgicale de la tumeur reste le traitement de choix, et l'examen histologique doit porter sur l'ensemble de la pièce d'exérèse, pour différencier un chondrome extra-osseux d'un chondrosarcome des parties molles bien différencié. Aucun cas de transformation maligne d'un chondrome extra-osseux n'a été décrit à ce jour. Malgré le caractère bénin de la lésion, une récidive locale survient dans 15 à 25% des cas [13, 14] mise sur le compte d'une exérèse incomplète ou en rapport avec un doute sur la nature histologique.

## Conclusion

Le chondrome des parties molles est une lésion rare, sa localisation au niveau de la cuisse est exceptionnelle. Le diagnostic est évoqué à l'examen clinique et radiologique, et confirmé à l'examen histologique. Une exérèse chirurgicale complète de la tumeur constitue le traitement de choix, et les récidives sont exceptionnelles.
